# The Impact of Socio-Demographic Factors on Breastfeeding: Findings from the “Mamma & Bambino” Cohort

**DOI:** 10.3390/medicina57020103

**Published:** 2021-01-24

**Authors:** Roberta Magnano San Lio, Andrea Maugeri, Maria Clara La Rosa, Antonio Cianci, Marco Panella, Giuliana Giunta, Antonella Agodi, Martina Barchitta

**Affiliations:** 1 Department of Medical and Surgical Sciences and Advanced Technologies “GF Ingrassia”, University of Catania, via S. Sofia, 87, 95123 Catania, Italy; roberta.magnanosanlio@phd.unict.it (R.M.S.L.); andrea.maugeri@unict.it (A.M.); mariclalarosa@gmail.com (M.C.L.R.); martina.barchitta@unict.it (M.B.); 2 Obstetrics and Gynecology Unit, Department of General Surgery and Medical Surgical Specialties, University of Catania, Via S. Sofia, 78, 95123 Catania, Italy; acianci@unict.it (A.C.); mpanella@unict.it (M.P.); giuntagiuliana.ct@gmail.com (G.G.)

**Keywords:** birth cohort, pregnancy, lactation, lifestyles, public health

## Abstract

*Background and objectives:* The World Health Organization (WHO) recommends women (1) to initiate breastfeeding within one hour of birth; (2) to exclusively breastfeed for the first six months; and (3) to continue breastfeeding until two years of age. However, women do not always adhere to these recommendations, threatening the health of their children. The present study aims to evaluate breastfeeding status and the main maternal factors associated with exclusive breastfeeding for six months among women from the “Mamma & Bambino” study, a prospective cohort settled in Catania, Italy. *Materials and Methods:* We used data from 220 women (median age = 37 years) enrolled in the “Mamma & Bambino” cohort during prenatal obstetric counselling. Self-reported breastfeeding status was collected during the follow-up interviews at 1 and 2 years, referring to breastfeeding status (i.e., yes or no) and type of breastfeeding (i.e., exclusive or predominant). We also collected data about duration of breastfeeding to classify women into those who adhered to the WHO recommendation and those who did not. *Results:* In the general population, we noted that the proportion of women who have breastfed increased with increasing educational level. Accordingly, logistic regression analysis demonstrated that medium (OR = 3.171; 95% CI = 1.285–7.822; *p* = 0.012) and high educational levels (OR = 4.549; 95% CI = 1.525–13.570; *p* = 0.007) were positively associated with breastfeeding if compared to low educational level. Among women who have breastfed, instead, the proportion of adherents to the WHO recommendation was higher among those with medium–high educational level and those who were employed. In line with this, we demonstrated that full-time employment (OR = 2.158; 95% CI = 1.033–4.508; *p* = 0.041) and medium educational level (OR = 4.632; 95% CI = 1.227–17.484; *p* = 0.024) were positively associated with exclusive breastfeeding for the first six months. *Conclusions:* Socio-demographic factors should be taken into account through public health strategies for improving maternal knowledge about health benefits of exclusive breastfeeding.

## 1. Introduction

Breastfeeding is one of the most effective ways to ensure child health and survival. Improving breastfeeding rates could save more than 820,000 children under five years every year [1]. Due to its importance, the World Health Organization (WHO) recommends that mothers should initiate breastfeeding within one hour of birth and infants should be breastfed exclusively for the first six months of life to achieve optimal growth. Moreover, the WHO suggests women to continue breastfeeding until two years of age of their newborns. However, worldwide, 7.6 million babies each year are never breastfed and nearly two out of three infants are not exclusively breastfed for the recommended six months [2]. The WHO defines exclusive breastfeeding as no other food or drink, not even water, except breast milk, while predominant breastfeeding requires breast milk as the primary source of nutrition, allowing for supplementation with liquids—including water and water-based drinks and fruit juice [3,4]. Breastmilk is considered the ideal food for infants due to containing a huge amount of nutrients—such as proteins, fats, sugars, vitamins, and minerals—that are perfectly balanced with each other to optimize their absorption in the intestine [5]. The long-term benefits of breastfeeding for mothers and children cannot be replicated with infant formula, which does not contain the antibodies, hormones, and growth factor that characterize breastmilk [6]. On the one hand, in babies, breastfeeding promotes healthy growth and supports healthy brain development with higher performance in intelligence tests [7]. On the other hand, breastfeeding protects mothers against postpartum depression, ovarian and breast cancer, heart disease, and type 2 diabetes [8].

For this reason, it is still relevant to develop public health strategies to promote breastfeeding practice among women and to raise their awareness about recommendations on exclusive breastfeeding and its proper timing. To identify mothers who could benefit from such public health interventions, however, it is necessary to understand what maternal characteristics might be associated with adherence to existing recommendations. Indeed, breastfeeding could be influenced by psychological and physiological factors, which, in turn, are related to a wide spectrum of environmental, socioeconomic, and cultural circumstances [9]. For instance, UNICEF reported that almost all newborns are breastfed in low-income countries, while one in five babies are never breastfed in high-income countries. Interestingly, in high-income countries, mothers from poorer households are less likely to breastfeed compared with their wealthier counterpart [7]. In line with this evidence, previous studies investigated the effects of maternal education and employment status on breastfeeding status and adherence to recommendations [10,11,12,13,14,15,16,17].

A review of sixteen Italian studies on breastfeeding concluded that published information depicted an inaccurate scenario about the prevalence and duration of breastfeeding in Italy. Inaccuracies depended on non-representative samples, the absence of standard definitions, and different recall periods [18]. To further explore this issue in Italy, we first evaluated the prevalence of breastfeeding and adherence to the WHO recommendations among women from the “Mamma & Bambino” study, a prospective cohort which enrolls mother–child pairs from Catania, Italy. Next, we investigated what maternal factors were associated with breastfeeding status and with the recommendation of exclusive breastfeeding for the first six months of life. We focused on this WHO recommendation, since it has probably the single largest potential impact of any preventive intervention [19].

## 2. Materials and Methods

### 2.1. Study Design

We used data from the “Mamma & Bambino” cohort, an ongoing Italian birth cohort whose study design and protocols are fully described elsewhere [20,21,22,23]. Further information can be also found at the website http://www.birthcohorts.net. The study protocol was approved by the ethics committees of the involved institutions (Ethics Committee of the “Azienda Ospedaliero-Universitaria Policlinico-Vittorio Emanuele” and Ethics Committee “Catania 1”; protocol numbers and date of approval: 47/2014/VE on 29 April 2014; 48/2015/EMPO on 20 April 2015; 186/2015/EMPO on 17 December 2015; 197/2016/EMPO on 21 December 2016; 213/2017/EMPO on 11 December 2017; 231/2018/EMPO on 10 December 2018; 263/2019/EMPO on 25 November 2019) and performed according to the Declaration of Helsinki. All women were fully informed of the purpose and procedures and gave their written informed consent. For mothers younger than 18 years, written informed consent was obtained by parents or a legal guardian. The study prospectively recruited pregnant women referred to the Azienda Ospedaliera Universitaria Policlinico “G. Rodolico-San Marco” (Catania, Italy) during prenatal obstetric counselling (mean gestational week of recruitment was 16th week) with planned follow-up until the second year of their child’s life. In this study, we included all mothers who completed pregnancy with a full assessment of information at the 2-year follow-up. By contrast, mothers with plurality, pre-existing medical conditions, and pregnancy complications were excluded.

### 2.2. Data Collection

Information on mothers and their children was collected through face-to-face interviews at recruitment, with planned telephone follow-ups at the delivery, and 1 and 2 years after birth. At the recruitment, a structured questionnaire was administered by trained epidemiologists to collect information on socio-demographic and behavioral factors. Educational level was categorized as low (primary school, i.e., ≤8 years of school), medium (secondary school, i.e., ≤13 years of school), and high education level (greater than 13 years of school). Women are also classified as full-time employed, part-time employed, and unemployed, which includes students and housewives. Smoking status was classified as no-smokers, former, and current smokers. Moreover, dietary data were collected using a 95-item semi-quantitative Food Frequency Questionnaire (FFQ), which referred to the previous month [24,25,26,27,28]. The adherence to Mediterranean diet (MD) was evaluated using the Mediterranean Diet Score (MDS) as previously described [26,29,30,31]. MDS ranged from 0 (non-adherence) to 9 (perfect adherence), and adherence was categorized as follows: low adherence (MDS range: 0–3), medium adherence (MDS range: 4–6), or high adherence (MDS range: 7–9) [32]. At the recruitment, we also asked women to report their pre-pregnancy weight and height to calculate pre-pregnancy body mass index (BMI) as weight in kilograms divided by height in meters squared. Pre-pregnancy BMI is classified according to WHO criteria [33]. Gestational weight gain (GWG) is classified as adequate, reduced, or excessive according to pre-pregnancy BMI and recommendations from the Institute of Medicine (IOM) [34]. At birth, information about type of delivery was collected through telephone interview, classifying women into those who had a natural birth and those who had a caesarean section. For the current analysis, we also used information regarding self-reported breastfeeding status, which were collected through telephone interviews at 1- and 2-year follow-up after birth. Specifically, these follow-up interviews were conducted through structured questionnaires and collect information on breastfeeding status (i.e., yes or no) at 1 and 2 years of life, date of starting and ending breastfeeding, type of breastfeeding (i.e., exclusive or predominant), and time of change from exclusive to predominant breastfeeding, if present. After checking for consistency between 1- and 2-year interviews, we comprehensively used this information to classify women into those who have breastfed and those who did not until the 2nd year of life of their child. Moreover, we defined women as those who have exclusively breastfed if their children have received only breast milk and no other liquids or solids for at least one month. During follow-up interviews, we also collect data on complementary feeding. Given that, we considered as outcomes the adherence to WHO recommendations on breastfeeding, which require (1) to initiate breastfeeding within the first hour of life, (2) to exclusively breastfeed for the first six months of life (i.e., meaning no other foods or liquid are provided), and (3) to continue breastfeeding receiving complementary foods until 2 years of age [2].

### 2.3. Statistical Analyses

Statistical analyses were performed using SPSS software version 26.0 (SPSS, Chicago, IL, USA). Prior to analysis, the normal distribution of continuous variables was checked using the Kolmogorov–Smirnov test. Descriptive statistics were used to describe maternal characteristics through frequency (%) for qualitative variables or median and interquartile range (IQR) for continuous variables due to their skewness. Accordingly, continuous variables underlying skewed distribution were compared across groups using the Mann–Whitney U test. Instead, binary and categorical variables were compared using the simple Chi-square test or the Chi-square test for trend, respectively. For categorical variables, we calculated the statistical power of our analyses using the Cochran–Armitage test for linear trend. Since statistical power depends on sample size, group weights, and probabilities of exposure, we separately calculated it for each exposure variable according to sample distribution. Specifically, the statistical power of our tests ranged from 69% to 97%, assuming a difference of 15% between alternative group probabilities of exposure. We also applied logistic regression analyses to identify main factors associated with breastfeeding status in the general population, and with adherence to the WHO recommendation of exclusive breastfeeding for 6 months in women who have breastfed. In both analyses, the logistic regression model simultaneously included educational level and employment status, which were associated with general breastfeeding status and/or with adherence to the WHO recommendation in univariate analyses. Both analyses were adjusted for maternal age and tested for interaction between educational level and employment status. Results were reported as Odds ratio (OR) and 95% confidence interval (CI). All statistical tests were two-sided, and *p*-values <0.05 were considered statistically significant.

## 3. Results

### 3.1. Characteristics of Study Population

**Figure 1:**
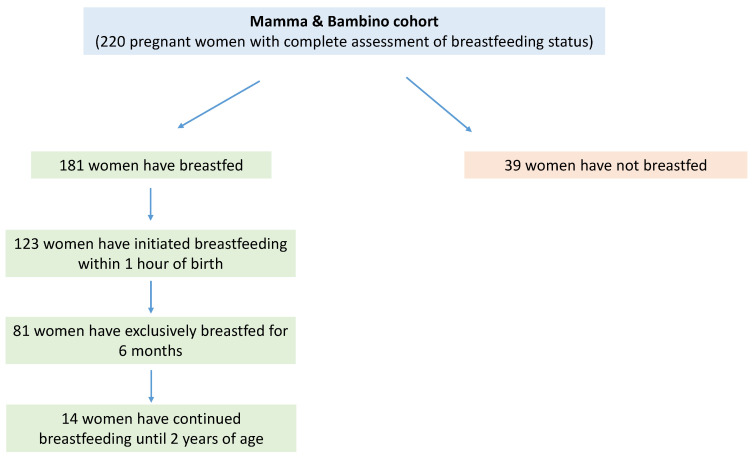
Distribution of women according to breastfeeding status and WHO recommendations.

The present analysis included 220 women (aged 15–45 years, median = 37 years) with a complete assessment of breastfeeding status from the “Mamma & Bambino” cohort. All women were enrolled at a median gestational age of 16 weeks from 2014 to 2018, and their characteristics are reported in Table 1. In brief, 66.7% of women had at least one child in addition to the one included in the present study. Moreover, 85.0% women reported a medium or high education level, while 58.2% were full-time or part-time employed. With respect to behavioral factors, 17.4% were current smokers and nearly 60.0% reported a medium adherence to MD. According to BMI (median = 22.7; IQR = 5), nearly a quarter of women (25.6%) were overweight or obese, with 37.0% of women with adequate GWG. Figure 1 shows the distribution of women according to breastfeeding status and WHO recommendations. Overall, 181 women declared to have breastfed. Specifically, we observed that 68.0% (*n* = 123) initiated breastfeeding within one hour of birth, out of which 65.9% have exclusively breastfed for the first 6 months (*n* = 81). However, only 7.7% (*n* = 14) of women who have breastfed continued until 2 years of age.

### 3.2. Association between Maternal Characteristics and Breastfeeding Status

Table 1 shows the characteristics of women according to their breastfeeding status. We observed an association between breastfeeding status and educational level (*p* = 0.001). Accordingly, logistic regression analysis demonstrated that medium (*p* = 0.012) and high educational level (*p* = 0.007) were positively associated with breastfeeding if compared with low educational level. By contrast, no association with employment status was evident (Table 2).

### 3.3. Association between Maternal Characteristics and Exclusive Breastfeeding for the First Six Months

We further compared maternal characteristics according to their adherence to the WHO recommendation of exclusive breastfeeding for the first six months (Table 3). Interestingly, we noted that the proportion of women who have exclusively breastfed for six months increased with increasing educational level (*p* = 0.018). Moreover, the proportion of women who adhered to this recommendation was higher among those who were employed than those who were unemployed (*p* = 0.015).

In line with these findings, we demonstrated that medium–high educational level and being employed were positively associated with exclusive breastfeeding for the first six months. Specifically, in the logistic regression model, full-time employed women (OR = 2.158; 95% CI = 1.033–4.508; *p* = 0.041) and those with medium educational level (OR = 4.632; 95% CI = 1.227–17.484; *p* = 0.024) were more likely to adhere to the WHO recommendation than their less educated and unemployed counterparts. We also observed borderline significant associations of part-time employment and high educational level with adherence to the WHO recommendation. (Table 4).

## 4. Discussion

Worldwide, human breast milk is recognized as the optimal source for infant nutrition due to its unique composition of microorganisms, metabolites, multipotent stem cells, growth factors, and other components. Thus, breastfeeding—also through molecular mechanisms—has clear short-term benefits on newborns and long-term effects during childhood and adolescence [1,35,36]. Similarly, long-term breastfeeding has been associated with health benefits for mothers [37,38,39]. In this scenario, the present study aims to uncover maternal factors associated with breastfeeding among 220 women from the “Mamma & Bambino” cohort. In this cohort, we found that 82.3% of women have breastfed, regardless of breastfeeding type. Interestingly, we observed that the proportion of women who have breastfed increased with increasing educational level. Our logistic regression analysis confirmed that medium and high educational levels were positively associated with breastfeeding, also considering maternal age and employment status. As suggested by previous studies [40,41], a plausible explanation of our findings is that women with a high educational level are more likely to seek medical advice and exploit health services with regard to breastfeeding [42]. Interestingly, different socioeconomic factors could also influence the duration of breastfeeding and its exclusivity.

From a public health perspective, it is important that newborns are breastfed exclusively during their first six months of life. However, compliance with this recommendation depends on several sociocultural factors [43]. In our study, nearly 64.0% of women declared to have exclusively breastfed for six months, according to the WHO recommendation. With respect to maternal characteristics, we noted that the proportion of women who adhered to this recommendation increased with increasing educational level. This result was confirmed by logistic regression analysis, in which women with a medium educational level were more likely to breastfeed exclusively for six months. Moreover, considering that the proportion of women who have exclusively breastfed for six months was higher among those employed, we demonstrated that full-time employment was positively associated with adherence to breastfeeding recommendations. We also observed borderline positive associations of part-time employment and high educational level with adherence to this recommendation. These findings could be explained by the positive effects that education has on breastfeeding knowledge in general, and on compliance with recommendations. With respect to employment status, it is important to consider the positive impact of Italian parental leave, which allows full-time and part-time employed women to continue exclusive breastfeeding for the recommended period. Considering socio-demographic factors (i.e., educational level and employment), recent evidence of their association with breastfeeding cessation before six months is scarce. A review conducted by Mangrio and colleagues [44] demonstrated that younger mothers with low educational level were more likely to interrupt breastfeeding before six months. In line with this, the study from Dubois and Girard [43] reported that the combined effects of age and educational level were so strong that other socioeconomic factors (i.e., family income and employment status) were not significant in the adjustment model. With respect to employment status, healthcare advisors and/or nurses should inform women how best to continue breastfeeding when returning to work. Six studies have been conducted to evaluate the association between returning to work within 12 weeks post birth and the cessation of breastfeeding before six months of life [10,11,12,13,14,15]. According to Tarrant and colleagues, women who started working early in the postpartum period were more likely to introduce infant formula, giving up exclusive breastfeeding [11]. Thus, time chosen to start working again and types of work could explain differences in adherence to breastfeeding recommendations among women.

Aside from socio-demographic determinants, breastfeeding could be affected by other factors. For instance, findings from studies conducted in developed countries stated an association between maternal pre-pregnancy obesity and higher risk of non-initiation and shorter breastfeeding duration [45,46]. Similarly, smoking during lactation was also associated with higher rates of early cessation, probably due to nicotine effects on milk volume and sleeping patterns [47,48]. However, in our study, we did not find a significant association between the abovementioned factors and breastfeeding.

In this scenario, breastfeeding programs should be implemented for all mothers, with specific interventions tailored toward less educated mothers. Particularly, socio-demographic factors should be considered through targeted interventions focusing on mothers who are at risk of interrupting breastfeeding before the recommended time. With this in mind, public health strategies are needed to identify cultural beliefs and practices that support infant feeding, in order to promote exclusive breastfeeding, which in turn affects maternal and child health.

Our study had some limitations. Firstly, the small size of our convenience sample did not allow us to completely exclude potential associations in those tests with low statistical power. This represents a plausible reason for the absence of association with factors that were previously related to breastfeeding by previous studies (e.g., pre-pregnancy BMI, smoking status, etc.). Moreover, the low number of women in some exposure categories (e.g., high educational level and part-time employment) might at least partially explain the borderline but not significant association observed in our study. The small sample size also did not allow us to generalize our findings and to adjust for additional potential confounders. In fact, along with socio-demographic determinants, breastfeeding could be affected by others factors, including maternal age [43], smoking [49,50,51,52], cesarean delivery [53,54,55,56], postpartum complications and breastfeeding difficulties [57,58], parity [53,55], and obesity [54,59,60]. Although many of them were evaluated in our study showing no association with breastfeeding, we cannot completely exclude the possibility of bias from residual unknown or unmeasured factors. Moreover, we considered socio-demographic information on mothers collected at recruitment, without taking into account any changes that occurred during the follow-ups at 1 and 2 years. For instance, it could be interesting to evaluate in the future whether women who return to work after maternity leave exhibit different compliance with breastfeeding recommendations [61,62]. Secondly, the proportion of infants who were breastfed until 2 years was low, not allowing us to determine what factors might be associated with prolonged breastfeeding with complementary feeding. Thirdly, data on breastfeeding were self-reported and collected through telephone interviews, which did not preclude potential reporting errors and may suffer from inaccuracies.

## 5. Conclusions

In spite of these limitations, however, our study underlined the need for strengthening breastfeeding programs. Indeed, 18% of women have never breastfed, 37% have exclusively breastfed for the first six months, and only 8% have continued to breastfeed until two years. With these numbers in mind, it should be necessary to develop strategies to improve mothers’ knowledge about breastfeeding benefits, but without blaming women that do not breastfeed for a wide variety of motives. In fact, the choice of breastfeeding certainly depends on the sociocultural background, but it could be precluded by psychological and physiological barriers.

## Figures and Tables

**Table 1:** Maternal characteristics according to breastfeeding practice.

Characteristics	Overall (*n* = 220)	Breastfeeding (*n* = 181)	Not Breastfeeding (*n* = 39)	*p*-Value
**Age (** **Years)**	37.0 (5.0)	37.0 (4.0)	38.0 (4.0)	0.656
**Educational Level**				
Low	33 (15.0%)	20 (11.0%)	13 (33.3%)	0.001
Medium	106 (48.2%)	89 (49.2%)	17 (43.6%)	
High	81 (36.8%)	72 (39.8%)	9 (23.1%)	
**Employment Status**				
Full-time employed	88 (40.0%)	77 (42.5%)	11 (28.2%)	0.240
Part-time employed	36 (16.4%)	29 (16.0%)	7 (17.9%)	
Unemployed	96 (43.6%)	75 (41.4%)	21 (53.8%)	
**Smoking Status**				
Current	38 (17.4%)	30 (16.8%)	8 (20.5%)	0.601
Former	52 (23.9%)	45 (25.1%)	7 (17.9%)	
Never	128 (58.7%)	104 (58.1%)	24 (61.5%)	
**Pre-Gestational BMI**	22.7 (4.5)	22.6 (4.5)	23.2 (4.9)	0.768
**Gestational Weight Gain ^a^**				
Reduced	86 (39.3%)	72 (40.0%)	14 (35.9%)	0.291
Adequate	81 (37.0%)	69 (38.3%)	12 (30.8%)	
Excessive	52 (23.7%)	39 (21.7%)	13 (33.3%)	
**Having Children ^b^**				
Yes	138 (66.7%)	115 (68.5%)	23 (59.0%)	0.258
No	69 (33.3%)	53 (31.5%)	16 (41.0%)	
**Adherence to Mediterranean Diet**				
Low	76 (34.7%)	61 (33.9%)	15 (38.5%)	0.638
Medium	131 (59.8%)	110 (61.1%)	21 (53.8%)	
High	12 (5.5%)	9 (5.0%)	3 (7.7%)	
**Type of Delivery**				
Natural	121 (57.3%)	100 (57.8%)	21 (55.3%)	0.774
Caesarean	90 (42.7%)	73 (42.2%)	17 (44.7%)	

Data are reported as median (interquartile range) or frequency (percentage) and were compared using the Mann–Whitney U test or the Chi-squared test. ^a^ Classified according to pre-gestational BMI and recommendations from the Institute of Medicine. ^b^ Having at least one child in addition to the one included in the present study.

**Table 2:** Association between educational level and employment status with breastfeeding practice.

Characteristics	OR	95% CI	*p*-Value
**Educational Level**			
Low	ref		
Medium	3.171	1.285–7.822	0.012
High	4.549	1.525–13.570	0.007
**Employment Status**			
Unemployed	ref		
Part-time employed	0.922	0.339–2.508	0.874
Full-time employed	1.206	0.489–2.977	0.684

Odds ratio (OR) and 95% Confidence Intervals (95% CI) are based on logistic regression model including both educational level and employment status and adjusting for maternal age (continuous).

**Table 3:** Characteristics of women who have breastfed, according to the WHO recommendation of 6-month exclusive breastfeeding.

Characteristics	Adherent (*n* = 81)	Non-Adherent (*n* = 100)	*p*-Value
**Age (** **Years)**	37.0 (4.0)	38.0 (4.0)	0.293
**Educational Level**			
Low	3 (3.7%)	17 (17.0%)	0.018
Medium	43 (53.1%)	46 (46.0%)	
High	35 (43.2%)	37 (37.0%)	
**Employment Status**			
Full-time employed	41 (50.6%)	36 (36.0%)	0.015
Part-time employed	16 (19.8%)	13 (13.0%)	
Unemployed	24 (29.6%)	51 (51.0%)	
**Smoking Status**			
Current	13 (16.3%)	17 (16.3%)	0.983
Former	20 (25.0%)	25 (25.3%)	
Never	47 (58.8%)	57 (57.6%)	
**Pre-gestational BMI**	22.3 (4.2)	22.7 (4.8)	0.236
**Gestational Weight Gain ^a^**			
Reduced	37 (46.3%)	35 (35.0%)	0.304
Adequate	27 (33.8%)	42 (42.0%)	
Excessive	16 (20.0%)	23 (23.0%)	
**Having Children ^b^**			
Yes	48 (66.7%)	67 (69.8%)	0.666
No	24 (33.3%)	29 (30.2%)	
**Adherence to Mediterranean Diet**			
Low	25 (30.9%)	36 (36.4%)	0.730
Medium	52 (64.2%)	58 (58.6%)	
High	4 (4.9%)	5 (5.1%)	
**Type of Delivery**			
Natural	45 (58.4%)	55 (57.3%)	0.879
Caesarean	32 (41.6%)	41 (42.7%)	

Data are reported as median (interquartile range) or frequency (percentage) and compared using the Mann–Whitney U test or the Chi-squared test. ^a^ Classified according to pre-gestational BMI and recommendations from the Institute of Medicine. ^b^ Having at least one child in addition to the one included in the present study.

**Table 4:** Association between educational level and employment status with adherence to the WHO recommendation of 6-month exclusive breastfeeding.

**Characteristics**	**OR**	**95% CI**	***p-*** **Value**
**Educational Level**			
Low	ref		
Medium	4.632	1.227–17.484	0.024
High	3.727	0.925–15.009	0.064
**Employment Status**			
Unemployed	ref		
Part-time employed	2.423	0.978–5.999	0.056
Full-time employed	2.158	1.033–4.508	0.041

Odds ratio (OR) and 95% Confidence Intervals (95%CI) are based on logistic regression model including both educational level and employment status and adjusting for maternal age (continuous).

## Data Availability

The data presented in this study are available on request from the corresponding author.
